# Selection of Reference Genes for Quantitative Gene Expression in Porcine Mesenchymal Stem Cells Derived from Various Sources along with Differentiation into Multilineages

**DOI:** 10.1155/2015/235192

**Published:** 2015-04-20

**Authors:** Won-Jae Lee, Ryoung-Hoon Jeon, Si-Jung Jang, Ji-Sung Park, Seung-Chan Lee, Raghavendra Baregundi Subbarao, Sung-Lim Lee, Bong-Wook Park, William Allan King, Gyu-Jin Rho

**Affiliations:** ^1^Department of Theriogenology and Biotechnology, College of Veterinary Medicine, Gyeongsang National University, 501 Jinju-daero, Jinju 660-701, Republic of Korea; ^2^PWG Genetics Pte Ltd., 15 Tech Park Crescent, Singapore 638117; ^3^Department of Oral and Maxilla Facial Surgery, School of Medicine, Gyeongsang National University, 501 Jinju-daero, Jinju 660-702, Republic of Korea; ^4^Department of Biomedical Sciences, University of Guelph, Guelph, ON, Canada N1G 2W1; ^5^Research Institute of Life Sciences, Gyeongsang National University, 501 Jinju-daero, Jinju 660-701, Republic of Korea

## Abstract

The identification of stable reference genes is a prerequisite for ensuring accurate validation of gene expression, yet too little is known about stable reference genes of porcine MSCs. The present study was, therefore, conducted to assess the stability of reference genes in porcine MSCs derived from bone marrow (BMSCs), adipose (AMSCs), and skin (SMSCs) with their in vitro differentiated cells into mesenchymal lineages such as adipocytes, osteocytes, and chondrocytes. Twelve commonly used reference genes were investigated for their threshold cycle (Ct) values by qRT-PCR. The Ct values of candidate reference genes were analyzed by geNorm software to clarify stable expression regardless of experimental conditions. Thus, Pearson's correlation was applied to determine correlation between the three most stable reference genes (NF_3_) and optimal number of reference genes (NF_opt_). In assessment of stability of reference gene across experimental conditions by geNorm analysis, undifferentiated MSCs and each differentiated status into mesenchymal lineages showed slightly different results but similar patterns about more or less stable rankings. Furthermore, Pearson's correlation revealed high correlation (*r* > 0.9) between NF_3_ and NF_opt_. Overall, the present study showed that* HMBS*,* YWHAZ*,* SDHA*, and* TBP *are suitable reference genes for qRT-PCR in porcine MSCs.

## 1. Introduction

Due to their physiological, anatomical, and genetic similarities with humans, porcine models are of great research interest. Porcine mesenchymal stem cells (MSCs) are not only in the spotlight for cell therapy, regenerative medicine, and tissue engineering [1, 2] but also as valuable research models for cellular and molecular understandings of optimal identification and multi-lineage differentiation.

MSCs are of great interest due to their self-renewal property, wide accessibility, multilineage differentiation potential into adipocytes, osteocytes, chondrocytes, neurons, and cardiomyocytes [3, 4] and immunomodulation by interfering with immune related cells [5]. These cells are found in various tissues such as bone marrow, dermal skin, adipose tissue, skeletal muscle, umbilical cord, hair follicles, synovial membrane, teeth, and placenta and these MSCs from different cell sources have revealed variant characteristics on proliferation, differentiation, and cell surface marker expression [6–9]. To verify the potential of MSCs by comparative characterization, quantitative PCR (qRT-PCR) analysis has been commonly considered as a tool for investigating gene expression. The qRT-PCR is a common technique used for measuring gene transcripts by amplified cDNA and widely employed due to its convenience, sensitivity, reproducibility, and reliability [10].

Reference genes are regarded as being involved in vital functions of cell survival with unchangeable expression regardless of experimental procedures or conditions [11–13]. However, recent studies have shown that, in some cells, genes considered as suitable reference genes have variable expression patterns in different tissues [14, 15]. Furthermore, during in vitro differentiation of porcine MSCs into specific lineages, intracellular and molecular features substantially change particularly with regard to regulations of transcription factors, metabolism, and cell proliferation [8]. Thus, validation of proper reference genes is a prerequisite for reliable results during characterization of MSCs by qRT-PCR.

Since the first finding of geometric averaging of multiple reference genes by algorithm for qRT-PCR analysis, the geNorm software [13], stable reference genes have been reported in various fields [15–20]. Stable porcine reference genes have been investigated under various conditions such as different tissues, age, and treatments in a variety of porcine cells [14, 15, 17–20]. However, little information on suitable reference genes is available for porcine MSCs. Moreover, the application of nonvalidated/unsuitable reference genes for normalization is likely to result in false conclusion confounding the understanding of the gene expression in MSCs [21, 22].

In this study, expression of twelve porcine reference genes which were used in previous studies [14, 15, 17–20] was compared to assess the stability regardless of cell status including undifferentiated porcine bone marrow derived MSCs (BMSCs), adipose derived MSCs (AMSCs), and skin derived MSCs (SMSCs) and their differentiated cells into adipocytes, osteocytes, and chondrocytes, respectively. The main objective of this research was to provide stable porcine reference genes for examining accurate relative mRNA fold changes at normalization in qRT-PCR data under undifferentiated and differentiated MSCs and we hypothesize that present reference genes are appropriate for accurately analyzing gene of interest in porcine MSCs.

## 2. Materials and Methods

### 2.1. Chemicals and Media

All chemicals were purchased from Sigma Aldrich (St. Louis, MO, USA) and media from Gibco (Invitrogen, Burlington, ON, Canada), unless otherwise specified.

### 2.2. Cell Isolation and Culture

All experiments were authorized by the Animal Center for Biomedical Experimentation at Gyeongsang National University. Bone marrow, subcutaneous adipose, and dermal tissues from ~1 year old male pigs (*n* = 5) were collected under standard procedures to isolate MSCs. MSCs from bone marrow extract were isolated by Ficoll (Ficoll Paque PLUS, GE Health care, Uppsala, Sweden) gradient method as previously described [3]. MSCs from subcutaneous adipose tissue were isolated by digestion with 0.1% collagenase type IV and subsequently separated by filtration with 100 and 40 *μ*m cell strainers. Dermal skin MSCs were minced and explanted onto culture dish as previously described [23]. In all kinds of MSCs isolation, Once the cells attached to the culture dish, supernatant was removed and changed with fresh culture media. All cells were cultured in advanced Dulbecco's Modified Eagle Medium (ADMEM) with 10% fetal bovine serum (FBS), 1% GlutaMax (Gibco), 10 ng/mL bFGF, and 1% penicillin-streptomycin (10,000 IU and 10,000 *μ*g/mL, resp., Pen-Strep) at 38.5°C in a humidified incubator at 5% CO_2_ in air. Once confluent, cells were trypsinized with 0.25% trypsin-EDTA solution, subcultured, and passaged 4 times before further analysis. All the experiments were repeated at least 3 times for data interpretation and statistical analysis.

### 2.3. Cell Surface Marker Analysis

All MSCs were analyzed for the presence of mesenchymal markers (CD29, CD44, CD90, and vimentin) and absence of hematopoietic marker (CD45) using a flow cytometer (BD FACS Calibur; Becton Dickinson, NJ, USA) as previously described [23]. Briefly, MSCs at ~80% confluence were harvested and fixed in 4% paraformaldehyde at 4°C for overnight. Fluorescein isothiocyanate (FITC) conjugated mouse rat anti-mouse CD44 (1 : 200; BD Pharmingen, BD Biosciences, Franklin Lake, NJ, USA), rat anti-mouse CD45 (1 : 200; Santa Cruz biotechnology, Inc., CA, USA), and mouse anti-human CD90 (1 : 200; BD Pharmingen) were labeled directly at room temperature for 1 h. Unconjugated mouse anti-pig CD29 (1 : 200; BD Pharmingen) and mouse anti-vimentin (1 : 200; Sigma) primary antibodies were incubated at room temperature for 1 h and followed by incubation with FITC-conjugated secondary antibodies (FITC goat anti-mouse IgG (1 : 200; BD Pharmingen)) at room temperature for 1 h. A total of 1 × 10^4^ FITC-labeled cells were counted by BD FACS Calibur.

### 2.4. In Vitro Differentiation and Confirmation

In vitro differentiation of all MSCs at 70~80% confluent was induced into adipocytes, osteocytes, and chondrocytes for 3 weeks under the specific conditions following previously described protocols [3, 24]. Adipogenic or osteogenic differentiation was induced in a culture medium supplemented with 100 *μ*M indomethacin, 10 *μ*M insulin, and 1 *μ*M dexamethasone or 200 *μ*M ascorbic acid, 10 mM *β*-glycerophosphate, and 0.1 *μ*M dexamethasone, respectively. For chondrogenic differentiation, STEMPRO Chondrogenesis Differentiation media with 10% supplement (Gibco) were used following manufacturer's protocol. Differentiated cells were then fixed with 4% paraformaldehyde and stained with 0.5% Oil red O solution, 5% silver nitrate solution (Von Kossa staining), or 1% alcian blue solution, respectively, to evaluate the accumulation of intracellular lipid droplets, calcium deposit, or proteoglycan. To confirm osteogenic differentiation, alkaline phosphatase (AP) activity was evaluated by staining with Western Blue Stabilized Substrate (Promega, USA) following manufacturer's instruction.

Differentiated cells in mesenchymal lineages were compared to undifferentiated MSCs with regard to the intensity of lineages specific gene expression by reverse transcription polymerase chain reaction (RT-PCR). Total mRNA isolation, cDNA synthesis, and RT-PCR amplification were carried out as in the previously described protocols [24]. RT-PCR program was comprised of predenaturation at 95°C for 10 min; 30–35 PCR cycles at 94°C for 30 s, 60°C for 30 s, and 72°C for 45 s; final extension 72°C for 10 min. Detailed information of primers for each lineage specific genes, lipoprotein lipase (*LPL*) and adipocytes fatty acid binding protein (*AP2*) for adipocytes, osteonectin (*ON*) and osteopontin (*OPN*) for osteocytes and aggrecan (*ACAN*), and collagen type X alpha 1 (*COL10A1*) for chondrocytes, is summarized in Table 1.

### 2.5. Candidate Reference Genes and Primer Sequences

Primers of twelve reference genes were selected in consideration of different intracellular biological function. Most of the primers were designed by Primer 3 Plus software and confirmed not to form hairpins, homodimers, and heterodimers by OligoAnalyzer 3.1 software. Primer sequences and information are presented in Table 1. Primer sequences of* GAPDH*,* TBP,* and* RPL4* were taken from Nygard et al. [14].

### 2.6. RNA Extraction, cDNA Synthesis, and qRT-PCR

Total RNAs were extracted from the MSCs before and after differentiation using RNeasy Mini Kit (Qiagen, CA, USA) according to the manufacturer's instructions and genomic DNA contamination was eliminated using RNase Free DNase (Qiagen) treatment for 15 min. Total RNAs were quantified using OPTIZEN 3220 UV BIO Spectrophotometer (Mecasys co., Ltd., Korea) and we selected pure total RNAs within 2 ± 0.2 of A260/A280 ratio. The reverse transcription of 1 *μ*g total RNAs to cDNA was synthesized using Omniscript Reverse Transcription Kit (Qiagen) with 1 *μ*M oligo dT primer (Invitrogen) at 60°C for 1 h. qRT-PCR was performed using Rotor Gene Q (Qiagen) qRT-PCR machine with Rotor-Gene 2X SYBR Green mix (Qiagen) including 1 *μ*M forward and reverse primers with 0.1 *μ*g cDNA per one reaction. The qRT-PCR program was comprised of predenaturation at 95°C for 10 min; 45 PCR cycles at 95°C for 10 s, 60°C for 6 s, and 72°C for 4 s; melting curve from 60°C to 95°C by 1°C per second; cooling at 40°C for 30 s according to qRT-PCR program with minor modification in manufacturer's protocol. Amplification curves, melting curves, and cycle threshold values (Ct values) were analyzed using Rotor-Gene Q Series Software (Qiagen). All PCR products were confirmed by electrophoresis using 1% agarose gel with 0.1 mg/mL ethidium bromide for nonspecific amplification with negative control. Images were analyzed using zoom browser EX5.7 software (Canon).

### 2.7. Analysis of Stable Reference Gene Expression

geNorm software was employed to analyze the stability of reference gene as previously described [13, 14]. The software evaluates stability measurement* M* (*M* values) by calculating the levels of average pairwise variation* V* for each gene with all other genes. Higher* M* values represent lower stable expression. After removal of a gene representing the highest* M* value from tested other genes sequentially, geNorm evaluated the ranking of stability continuously till the two most stable genes remained. For determining acceptable expression of each gene, 1.5 of* M* value was the cut-off value in analysis [13]. Furthermore, geNorm also evaluates the optimal number of reference genes by the normalization factor (NF_*n*_) from calculating the geometric mean. Pairwise variation *V*_*n*/*n*+1_ between two sequential NF_*n*_ and NF_*n*+1_ is estimated by continuous calculation depending on adding other genes sequentially from two genes at the lowest* M* value. Estimated large variation of the added gene indicates a substantial effect at the normalization and needs addition for calculation of a reliable normalization factor preferably. Correlations of normalization factor between the optimal number of reference genes (NF_opt_) and the three most stable reference genes (NF_3_) were analyzed by Pearson's correlation in SPSS. In the present study, the application of geNorm is programmed to list the order of stable reference genes from different MSCs before and after differentiation into mesenchymal lineage cells. Experimental groups for analysis of stable reference gene expression were allocated into five experimental groups: undifferentiated MSCs from different sources; those MSCs before and after differentiation into adipocytes, osteocytes, and chondrocytes, respectively; those MSCs before and after differentiation into all mesenchymal lineages.

### 2.8. Statistical Analysis

Ct values were presented as means ± SEM and analyzed using one-way ANOVA and Tukey post hoc test. Significant differences were considered at *P* < 0.05.

## 3. Result

### 3.1. Morphology and Cell Surface Markers

**Figure 1:**
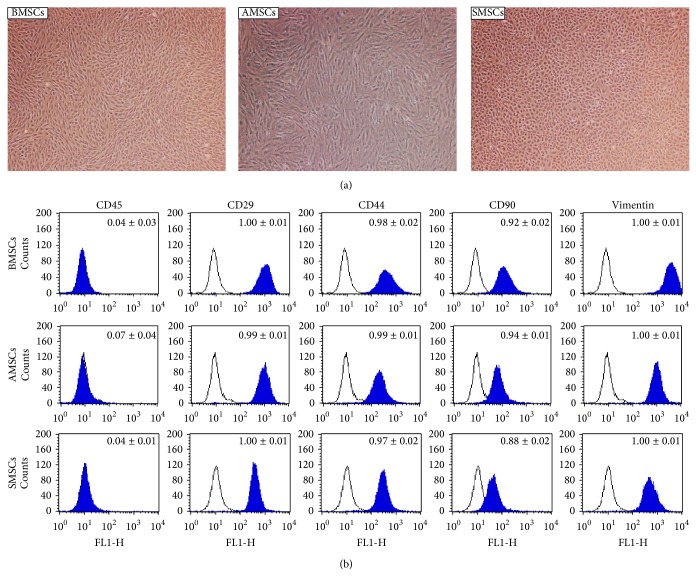
Morphology of MSCs and cell surface antigen expression. BMSCs, AMSCs, and SMSCs were cultured in separate 35 mm dishes and displayed the morphology at 4 passages in phase contrast images (a). Three kinds of MSCs were analyzed for MSCs specific surface antigen expression by flow cytometry (b). Open histograms imply isotype IgG expression as control, and filled histograms represent each surface antigen expression. Mean% ± SD of each MSCs expression in respective three biological replications was presented on top of each graph. BMSCs: bone marrow derived mesenchymal stem cells; AMSCs: adipose derived mesenchymal stem cells; SMSCs: skin derived mesenchymal stem cells. Magnification ×100.

MSCs isolated from bone marrow, subcutaneous adipose, and dermal tissues were observed to have similar fibroblastic morphologies with dendritic spindle shapes and grew as adherent cells in culture dishes (Figure 1(a)). MSCs from all tissue types expressed MSCs specific markers, such as CD29, CD44, CD90, and vimentin, whereas CD45, a hematopoietic stem cell marker, was negative in all MSCs as determined by FACS analysis as shown in Figure 1(b).

### 3.2. In Vitro Differentiation

**Figure 2:**
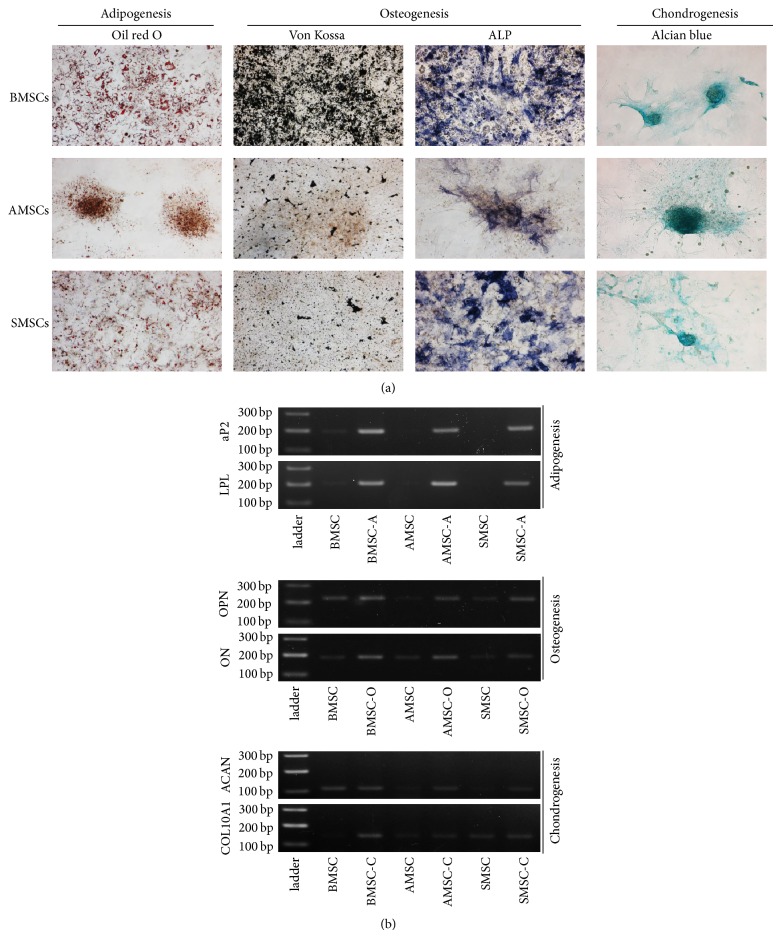
Confirmation of differentiation ability into various mesenchymal lineages. Three kinds of MSCs were differentiated into adipocytes, osteocytes, and chondrocytes for three weeks. (a) Cytochemical staining of each differentiated MSCs with Oil red O, Von Kossa, AP staining, and alcian blue. (b) Increased intensities of lineage specific gene expression before and after differentiation in each MSCs by RT-PCR. Lanes, which were displayed from the left to the right, showed 100 bp or 200 bp ladder, undifferentiated MSCs, and differentiated cells in numerical order of BMSCs, AMSCs, and SMSCs, respectively.* AP2*: adipocyte fatty acid binding protein;* LPL*: lipoprotein lipase;* OPN*: osteopontin;* ON*: osteonectin;* ACAN*: aggrecan;* COL10A1*: collagen, type X, alpha 1; BMSC-A, BMSC-O, and BMSC-C: differentiated BMSC into adipocytes, osteoblasts, and chondrocytes, respectively; AMSC-A, AMSC-O, and AMSC-C: differentiated AMSC into adipocytes, osteoblasts, and chondrocytes, respectively; SMSC-A, SMSC-O, and SMSC-C: differentiated SMSC into adipocytes, osteoblasts, and chondrocytes, respectively. Magnification ×100.

MSCs from all types of tissue examined were successfully differentiated into mesenchymal lineages, adipocytes, osteocytes, and chondrocytes under the specific induction conditions. Cytochemistry was performed to evaluate differentiation and revealed that MSCs from all types of tissue made steady progress towards differentiating into adipocytes, osteocytes, and chondrocytes as conformed by Oil red O, Von Kossa, and alcian blue staining, respectively. Moreover, alkaline phosphatase activities were detected in osteogenic induced MSCs (Figure 2(a)). Furthermore, RT-PCR was performed to confirm the differentiation by assessing lineage specific gene expression, and amplified products of each gene in agarose gel were displayed in Figure 2(b).

### 3.3. Examination of Primer Specificity and Amplicon Size

**Figure 3:**
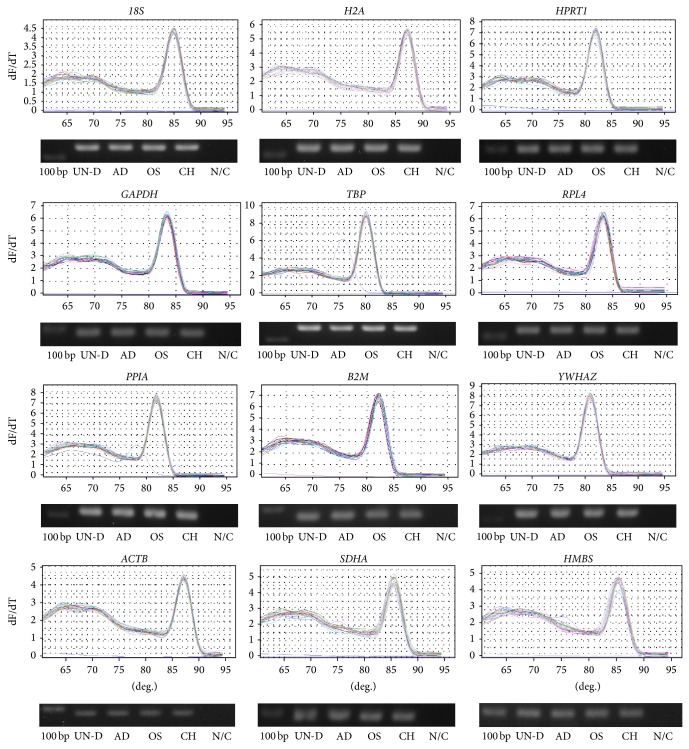
Confirmation of primer specificity and amplicon size in various MSCs with selected reference genes. Each melting curve analysis of twelve selected reference genes was presented (top of individual genes) and each product of amplification was shown by agarose gel electrophoresis (bottom of individual genes). Lanes, which were displayed from the left to the right, showed 100 bp ladder, undifferentiated MSCs, and differentiated cells into adipocytes or osteoblasts or chondrocytes, respectively. UN-D: undifferentiated MSCs; AD: adipogenesis; OS: osteogenesis; CH: chondrogenesis; N/C: negative control.

All candidates of reference genes were examined for primer specificity and nonspecific amplifications by melting curve analysis. All reference genes were amplified with a high peak of single products and without nonspecific amplification in melting curve analysis (Figure 3). In addition, all amplicons were confirmed as specific amplifications with appropriate product size by agarose gel electrophoresis, and agarose gel images were displayed at the bottom of each reference gene melting curve.

### 3.4. Average of Ct Values of Reference Genes in MSCs before and after Differentiation

**Figure 4:**
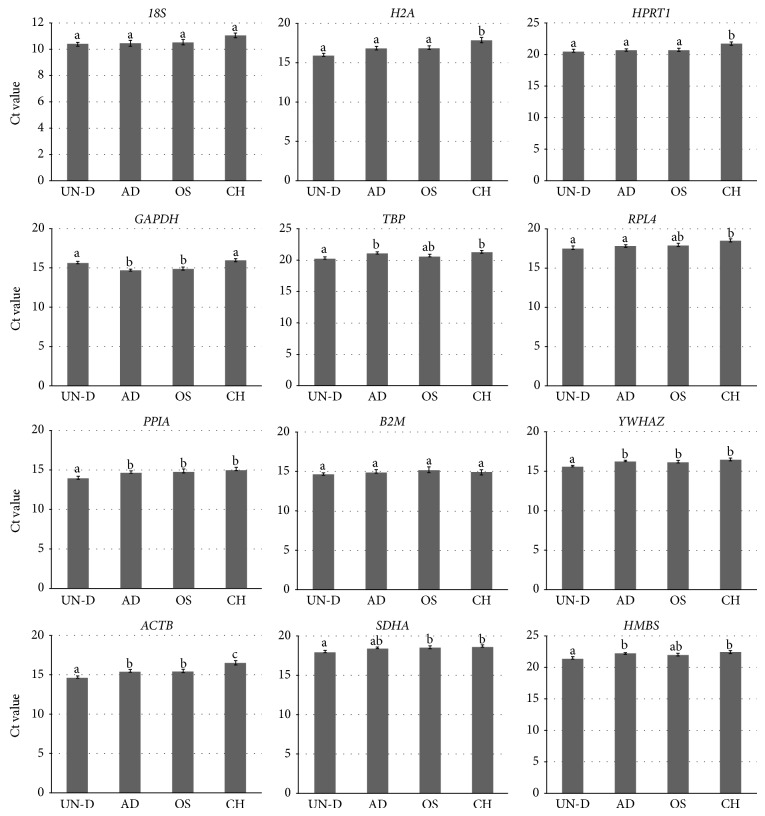
Average of Ct values in selected reference genes under various MSCs status. The Ct value of each primer was analyzed by ANOVA with Turkey's post hoc test. Significant differences were displayed by letters in the top of bars (*P* < 0.05). Graphs were presented as mean ± SEM.

Twelve reference genes were evaluated for changes in their mRNA levels among undifferentiated and differentiated MSCs by qRT-PCR. Cycle threshold (Ct) values of each selected reference gene were detected with significant differences, as shown in Figure 4. Ct values of* 18S* and* B2M* did not differ in MSCs before and after differentiation into mesenchymal lineages. However, the values of the other ten reference genes were significantly (*P* < 0.05) different in MSCs before and after differentiation. In particular,* H2A*,* HPRT1,* and* RPL4* showed significantly (*P* < 0.05) higher Ct values indicating low transcription levels in chondrogenesis.* ACTB*,* TBP*,* PPIA*,* YWHAZ*,* SDHA,* and* HMBS* showed significantly (*P* < 0.05) higher Ct value indicating low transcription levels in differentiated cells compared to undifferentiated MSCs.

### 3.5. Examination of Stable Reference Genes

**Figure 5:**
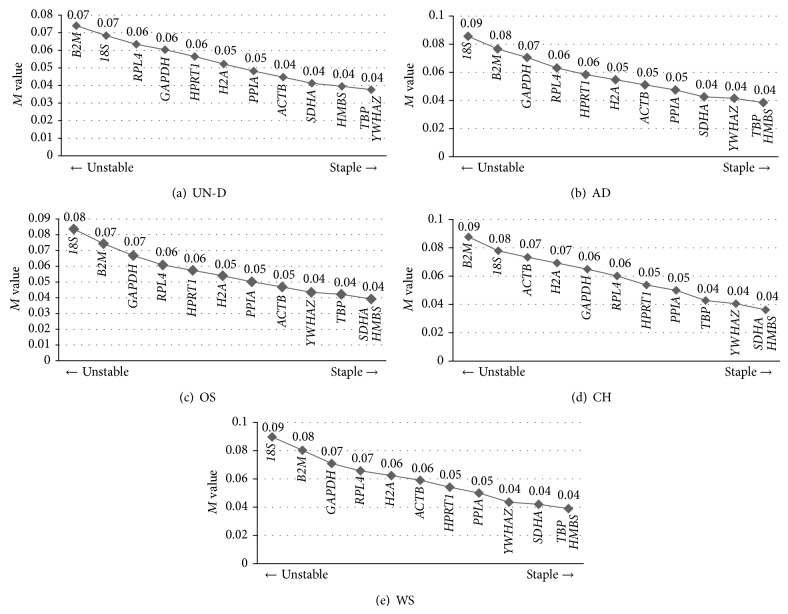
Ranking of stability (M values) in selected reference genes by using geNorm software. M values were analyzed from each Ct value categorizing five groups by geNorm software by calculating the average of pairwise variation between tested twelve reference genes. The left side of graph indicates high* M* value, which means low stability, and the right side of graph presents high stability with low* M* value. Each* M* value is presented above gene symbols.* M* values of twelve genes for (a) undifferentiated status of BMSCs, AMSCs, and SMSCs and (b–d) differentiated cells into adipocytes, osteocytes, and chondrocytes, respectively. (e) Whole status of MSCs.

The raw Ct values of MSCs before and after differentiation were analyzed using geNorm software program, which not only estimates the stability measure (*M* value) but also evaluates stable ranking of twelve candidate reference genes.* M* values are presented in Figure 4 with lower* M* values considered more stable [13]. All* M* values were lower than 1.5; therefore, all investigated genes were judged to have stable gene expression. Although rankings from five experimental groups showed slightly different results, there were similar patterns concerning stability of reference genes. In particular, various combinations of* HMBS*,* TBP*,* YWHAZ,* and* SDHA* were shown to be the more stable reference genes in all of five experimental groups (Figure 5(a)). In undifferentiated MSCs and adipogenesis,* HMBS*,* YWHAZ,* and* TBP* were revealed to have high stability (Figures 5(a) and 5(b)). In addition,* HMBS*,* SDHA,* and* TBP* were estimated to have high stability in osteogenesis and MSCs (Figures 4(c) and 4(e)). And also,* HMBS*,* SDHA,* and* YWHAZ* showed high stability in chondrogenesis (Figure 5(d)). However* 18S*,* GAPDH,* and* ACTB*, which are commonly used reference genes, displayed high* M* values and showed inadequate reference genes for qRT-PCR in various differentiated statuses of MSCs.

### 3.6. Examination of Optimal Number of Reference Genes and Pearson's Correlation

**Figure 6:**
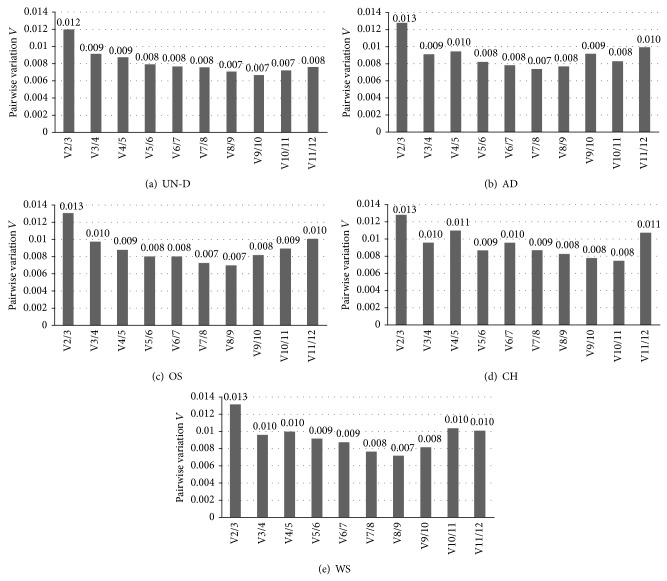
Optimal number of reference genes for normalization. Determining optimal number of reference genes is based on pairwise variation *V*_*n*/*n*+1_ by calculating each two sequential normalization factors. The low* V* value indicates optimal number of reference genes normalized to target genes. Each* V* value is presented above bars.* V* values for (a) undifferentiated status of BMSCs, AMSCs, and SMSCs and (b–d) differentiated cells into adipocytes, osteocytes, and chondrocytes, respectively. (e) Whole status of MSCs.

geNorm analysis determined not only stable reference genes presenting lower* M* values but also optimal numbers of reference genes for normalization by calculating the pairwise variation (*V*) indicating that lower values have less variation. All pairwise variations (*V*) in five experimental groups are presented in Figure 6. In this study, geNorm suggested that 7–10 reference genes were determined as an adequate number for accurate normalization. Undifferentiated MSCs (*V*_9/10_;* V* = 0.007) were recommended for use of 9 reference genes, and 7, 8, or 10 reference genes were evaluated for lower pairwise variation* V* in adipogenesis (*V*_7/8_;* V* = 0.007), osteogenesis (*V*_8/9_;* V* = 0.007), or chondrogenesis (*V*_10/11_;* V* = 0.008) for normalization in research of gene expression, respectively. Application of 8 reference genes for normalization was proposed in whole status of MSCs (*V*_8/9_;* V* = 0.007).

**Figure 7:**
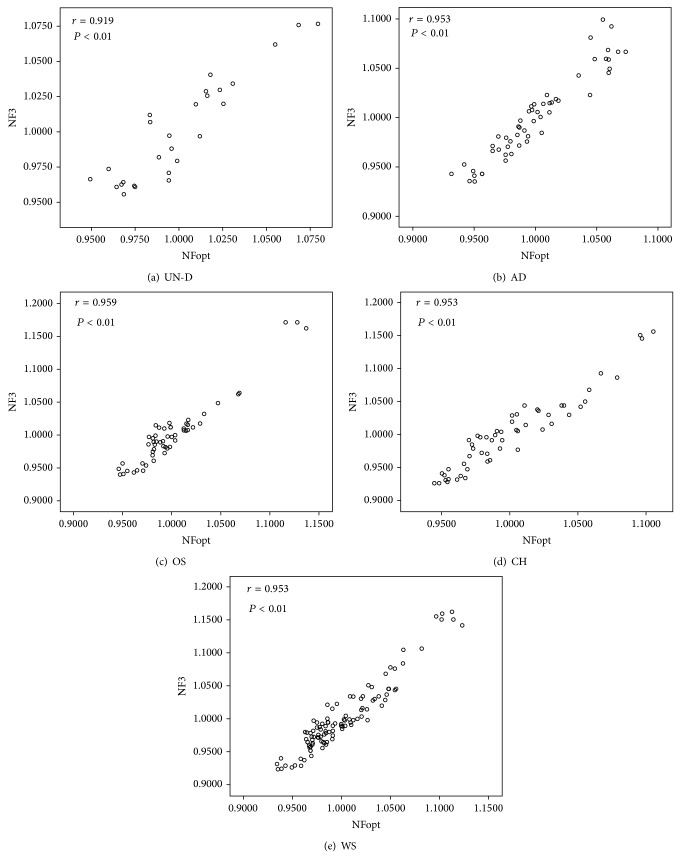
Correlation between NF_3_ and NF_opt_ by using Pearson's correlation. Pearson's correlation was applied between the three most stable reference genes at the lowest* M* value and optimal number of reference genes in the lowest pairwise variation* V* by geNorm software. The value about* r* > 0.9 (*P* < 0.01) indicates high correlation between two groups. Pearson's correlation for (a) undifferentiated status of BMSCs, AMSCs, and SMSCs and (b–d) differentiated cells into adipocytes, osteocytes, and chondrocytes, respectively. (e) Whole status of MSCs. UN-D: undifferentiated MSCs, AD: adipo differentiated, OS: osteo differentiated, CH: chondro differentiated, and WS: whole status.

However, it is grossly inefficient to apply 7–10 reference genes for normalization of target genes. Given this kind of inefficiency, we analyzed the correlation of normalization factor (NFs) between the three most stable reference genes (NF_3_) which corresponded to the three lowest* M* values and optimal number of reference genes (NF_opt_) presented in the lowest pairwise variation* V* for employment of the appropriate number of reference genes in each groups. As shown in Figure 7, NF_3_ and NF_opt_ were analyzed by Pearson's correlation and the result showed that all experimental groups had high correlation (*r* > 0.9, Pearson) between NF_3_ and NF_opt_. In these results, the three most stable reference genes of each group were sufficient for normalization at analyzing target genes in undifferentiated MSCs and differentiated cells into mesenchymal lineages.

## 4. Discussion 

In the present study, porcine BMSCs, AMSCs, and SMSCs were characterized by expression of MSCs specific surface antigen expression (CD29, CD44, CD45, CD90, and vimentin) and differentiation ability into adipocytes, osteocytes, and chondrocytes by confirmations of cytochemical staining (Oil red O, Von Kossa, alkaline phosphatase, and alcian blue) and lineage specific gene expression (*AP2, LPL, ON, OPN, ACAN,* and* COL10A1*). After induction of differentiation into mesenchymal lineages, all Ct values of each reference gene from undifferentiated and differentiated MSCs were investigated for assessment of stability ranking by geNorm software. Optimal numbers of reference genes (NF_opt_) were compared with three of the most stable reference genes (NF_3_) by Pearson's correlation for application of appropriate number of reference genes.

Selection of reference gene is indispensable step for evaluating relative mRNA expression by qRT-PCR, and selected reference genes should have constantly stable expression regardless of cell status, experimental procedure, and cell source [25, 26]. Attempts have been made to assess these requisites for determining various patterns of stability in reference genes under experimental conditions, such as different tissues, age, cell sources, and treatments [14, 15, 17–20]. The choice of validated reference genes at each experimental condition is an essential prerequisite for accurate normalization in qRT-PCR since selection of inappropriate reference gene leads to contradictable results [21].

Therefore, the present study compared the stability of twelve commonly used porcine reference genes to investigate stability of expression in porcine MSCs. MSCs expressed MSC specific surface antigens and could be differentiated into broad lineages, not only mesenchymal lineages such as adipocytes, osteocytes, and chondrocytes but also different lineages like hepatocytes and neuronal cells under suitable induction media [2–4, 9]. In this study, three kinds of MSCs negatively expressed CD45 but were positive for CD29, CD44, CD90, and vimentin, and successfully differentiated into adipocytes, osteocytes, and chondrocytes as confirmed by cytochemical staining. Differentiated cells were compared with undifferentiated MSCs for lineage specific markers such as* AP2* and* LPL* in adipogenesis,* ON* and* OPN* during osteogenesis, and* ACAN* and* COL10A1* in chondrogenesis [24, 27]. Even though MSCs have common characteristics, differentiation ability could be varied following various tissue sources. It is well known that BMSCs possessed stronger ability for osteogenesis than AMSCs and SMSCs for the deposition of mineral formation [3, 28]. In accordance with previous findings, oil droplet in adipogenesis and proteoglycan in chondrogenesis were detected positively across various differentiated MSCs, whereas dominantly osteogenic potential of BMSCs was observed by enhanced calcium deposit compared to other MSCs.

Using Ct value analysis, significant (*P* < 0.05) differences in ten reference genes were evaluated and these findings indicated the possibility that the expression patterns of each reference gene could be changed during the differentiation process (Figure 4). This appearance of significant differences in Ct values among experimental groups was previously reported in study of Ct values from different porcine tissues [14]. And various Ct value patterns of reference genes were also examined from fourteen different porcine tissues of different ages [15]. Interestingly, although* SDHA*,* PPIA*,* YWHAZ,* and* RPL4* had significantly different Ct values in fourteen different tissues,* M* values of these four genes by geNorm analysis were revealed to have more stable expression than other reference genes. On the contrary,* HPRT1* had the least significant differences in Ct values at fourteen different tissues but was determined to have less stable expression [15]. In agreement with these findings, our results also highlighted the inconsistency of reliability between Ct values and* M* values in MSCs. Although expression level of* 18S* and* B2M* showed no significant difference in Ct values in MSCs before and after differentiation,* M* values of* 18S* and* B2M* were judged to be less stable. Among twelve reference genes,* TBP*,* YWHAZ*,* SDHA,* and* HMBS* were examined and each showed more stable* M* values in spite of having different Ct values under differentiation induction (Figures 3 and 4).

A survey of the most frequently used reference genes by qRT-PCR, semi-qRT-PCR, and northern blotting through NCBI-PubMed proposed that* GAPDH* (27.24%),* ACTB* (30.62%), and* 18S* (12.52%) are ascertained as the three most widely used reference genes [18]. However, it has been verified that these three genes are not stable reference genes in porcine experiments. In particular,* GAPDH* revealed unstable gene expression in different tissues [14, 18, 29], ages [15], heat stressed peripheral blood mononuclear cells (PBMC) [30], and alveolar macrophage stimulations [17].* ACTB* showed irregular stability in different tissues [14] and moderate stability in mechanically impacted porcine articular cartilage [31] but showed unstable expression in different tissues, heat stressed PBMC, and alveolar macrophage stimulations [17, 18, 30]. Moreover, these three genes had irregular stabilities in different tissues after feeding diverse iodine concentrations [32]. In the present study,* GAPDH*,* ACTB,* and* 18S* were also determined as less stable reference genes. On the contrary,* HMBS*,* TBP*,* YWHAZ,* and* SDHA* were evaluated as more stable reference genes in all five experimental groups but the stable ranking in four genes was slightly different following each group (Figure 4). Similar to our observations,* TBP* was proposed to be the stable reference gene in different porcine tissues [14], heat stressed porcine PBMC [30], and porcine synovium derived MSCs cultured with two kinds of scaffolds system [20].* YWHAZ* was also expressed stably in different aged pigs [15] and porcine tissues [18]. Briefly, human MSCs also represented great changes of gene expression under differentiation induction [33]. And* TBP* and* YWHAZ* were determined as stable expression in undifferentiated AMSCs and BMSCs; furthermore, both genes were expressed stably in adipogenesis and chondrogenesis [34].

By calculating pairwise variation* V* values, 0.15 is recommended as cut-off value, below which the inclusion of an additional reference gene is not required [13]. All pairwise variation* V* values in this study were under 0.15; therefore, optimal numbers of reference genes (NF_opt_) presenting the lowest pairwise variation* V* were determined as 7–10 in each experimental group (Figure 5). In brief,* V*_9/10_ (this number means the pairwise variation of normalization factor between ninth and tenth reference genes and the number in front of slash means optimal number of reference genes) was presented in undifferentiated MSCs.* V*_7/8_ or* V*_10/11_ was proposed in adipogenesis or chondrocytes, respectively, as optimal number of reference genes. And* V*_8/9_ was estimated in osteogenesis and whole status of MSCs. In agreement with another research, because it is hard to use 7–10 reference genes for normalization of target genes, correlations of normalization factors were analyzed between optimal number (NF_opt_) and three of the most stable reference genes (NF_3_) by Pearson's correlation (Figure 6) were identified. Following the analysis, there were high correlations between NF_opt_ and NF_3_ (*r* > 0.9) in all experimental groups, and these results demonstrated that the use of three of the most stable reference genes for normalization would be satisfactory.

The use of the stable reference gene regardless of experimental procedures is necessary to evaluate precise target gene expression by qRT-PCR for ensuring reliable and reproducible results. The present study was conducted to find out stable reference gene in MSCs before and after differentiation, and* HMBS*,* YWHAZ*,* SDHA,* and* TBP* are proposed as suitable reference genes for qRT-PCR in porcine MSCs. These results are expected to play role in comparison to various properties of MSCs and their differentiated status for comparative characterization.

## Figures and Tables

**Table 1:** Sequence of primers used for candidate of reference genes and the evaluation of lineage specific genes.

Gene name (symbol)	Primer sequences	Amplicon size (bp)	Accession number or reference
18S ribosomal RNA (*18S*)	F: cgcggaaggatttaaagtg R: aaacggctaccacatccaag	141	AY265350.1

H2A histone family, member Z (*H2A*)	F: ggtaaggctgggaaggactc R: catggctggtcgtcctagat	124	NM_001123122.1

Hypoxanthine phosphoribosyltransferase1 (HPRT1)	F: aagcttgctggtgaaaagga R: gtcaagggcatagcctacca	100	NM_001032376.2

Glyceraldehyde-3-phosphate dehydrogenase (*GAPDH*)	F: acactcactcttctacctttg R: caaattcattgtcgtaccag	90	Nygard et al., 2007 [14]

TATA box binding protein (*TBP*)	F: aacagttcagtagttatgagccaga R: agatgttctcaaacgcttcg	153	Nygard et al., 2007 [14]

Ribosomal protein 4 (*RPL4*)	F: caagagtaactacaaccttc R: gaactctacgatgaatcttc	122	Nygard et al., 2007 [14]

Peptidylprolyl isomerase A (*PPIA*)	F: aaaacttccgtgctctgagc R: ttatggcgtgtgaagtcacc	112	NM_214353.1

Beta-2-microglobulin (*B2M*)	F: tccgccccagattgaaattg R: tccttgctgaaagacaggtctg	81	NM_213978.1

Tyrosine 3-monooxygenase/tryptophan 5-monooxygenase activation protein, zeta polypeptide (*YWHAZ*)	F: tgcttcctttgcttgcatcc R: tcagggtaggcagggtttatag	113	XM_001927228.4

Beta actin (*ACTB*)	F: tcaacaccccagccatgtac R: agtccatcacgatgccagtg	84	XM_003124280.2

Succinate dehydrogenase complex, subunit A (*SDHA*)	F: cacacgctttcctatgtcgatg R: tggcacagtcagcttcattc	94	XM_003362140.1

Hydroxymethylbilane synthase (*HMBS*)	F: ttcattccctcaaggacctg R: ggggtgaaagacaacagcat	101	NM_001097412.1

Adipocyte fatty acid binding protein (*AP2*)	F: aacccaacctgatcatcactg R: tctttccatcccacttctgc	192	AF102872.1

Lipoprotein lipase (*LPL*)	F: caaacttgtggctgccctat R: aaggctgtatcccaggaggt	202	Kumar et al., 2007 [24]

Osteonectin (*ON*)	F: tccggatctttcctttgctttcta R: ccttcacatcgtggcaagagtttg	187	Kumar et al., 2007 [24]

Osteopontin (*OPN*)	F: actccgatgaatccgatgag R: tccgtctcctcactttccac	220	Kumar et al., 2007 [24]

Aggrecan (*ACAN*)	F: agtggattggcttgaacgac R: agtggcgaagaagttgtcag	113	NM_001164652.1

Collagen, type X, alpha 1 (*COL10A1*)	F: gcaaacatgctgccacaaac R: gatgaagaactgtgccttggtg	141	NM_001005153.1
